# *N*-[1-(1*H*-Pyrrol-2-yl)ethyl­idene]aniline

**DOI:** 10.1107/S1600536812037695

**Published:** 2012-09-08

**Authors:** Bi-Yun Su, Lei Li, Jia-Xiang Wang, Xuan-Yan Li

**Affiliations:** a College of Chemistry and Chemical Engineering, Xi’an ShiYou University, Xi’an, Shaanxi 710065, People’s Republic of China

## Abstract

There are two independent mol­ecules in the asymmetric unit of the title compound, C_12_H_12_N_2_, in which the pyrrole and benzene rings form dihedral angles of 72.37 (7) and 82.34 (8)°. The imino N—C bond lengths in the two mol­ecules are equal [1.286 (2) Å] and indicate C=N character. In the crystal, each mol­ecule forms a dimer with an inversion-related mol­ecule through a pair of classical N—H⋯N hydrogen bonds.

## Related literature

For general background to the imino­pyrrole unit, see: Small *et al.* (1998 ▶); Su *et al.* (2009*a* ▶,*b* ▶); Britovsek *et al.* (2003 ▶); Dawson *et al.* (2000 ▶). For the pyrrole diimine unit, see: Matsuo *et al.* (2001 ▶) and for the pyrrole monoimine unit, see: He *et al.* (2009 ▶).
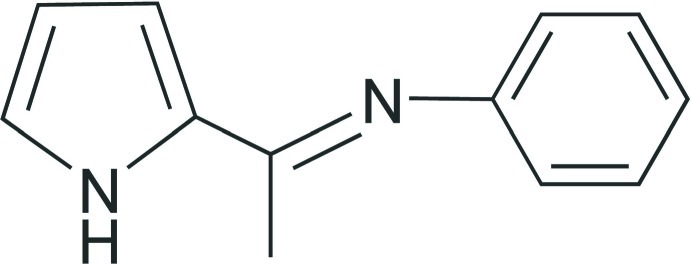


## Experimental

### 

#### Crystal data

C_12_H_12_N_2_
*M* *_r_* = 184.24
Triclinic, 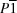

*a* = 8.2236 (14) Å
*b* = 11.3306 (19) Å
*c* = 11.913 (2) Å
α = 95.984 (3)°
β = 93.202 (3)°
γ = 109.274 (3)°
*V* = 1037.3 (3) Å^3^
*Z* = 4
Mo *K*α radiation
μ = 0.07 mm^−1^
*T* = 296 K
0.37 × 0.25 × 0.19 mm

#### Data collection

Bruker APEXII CCD diffractometer
Absorption correction: multi-scan (*SADABS*; Bruker, 2008 ▶) *T*_min_ = 0.974, *T*_max_ = 0.987
5277 measured reflections
3655 independent reflections
2567 reflections with *I* > 2σ(*I*)
*R*_int_ = 0.023

#### Refinement

*R*[*F* ^2^ > 2σ(*F* ^2^)] = 0.048
*wR*(*F* ^2^) = 0.142
*S* = 1.10
3655 reflections
256 parameters
H-atom parameters constrained
Δρ_max_ = 0.20 e Å^−3^
Δρ_min_ = −0.13 e Å^−3^

### 

Data collection: *APEX2* (Bruker,2008 ▶); cell refinement: *SAINT* (Bruker,2008 ▶); data reduction: *SAINT*; program(s) used to solve structure: *SHELXS97* (Sheldrick, 2008 ▶); program(s) used to refine structure: *SHELXL97* (Sheldrick, 2008 ▶); molecular graphics: *SHELXTL* (Sheldrick, 2008 ▶); software used to prepare material for publication: *publCIF* (Westrip, 2010 ▶).

## Supplementary Material

Crystal structure: contains datablock(s) I, global. DOI: 10.1107/S1600536812037695/rk2378sup1.cif

Structure factors: contains datablock(s) I. DOI: 10.1107/S1600536812037695/rk2378Isup2.hkl

Supplementary material file. DOI: 10.1107/S1600536812037695/rk2378Isup3.cml

Additional supplementary materials:  crystallographic information; 3D view; checkCIF report

## Figures and Tables

**Table 1:** Hydrogen-bond geometry (Å, °)

*D*—H⋯*A*	*D*—H	H⋯*A*	*D*⋯*A*	*D*—H⋯*A*
N1—H1*A*⋯N2^i^	0.86	2.38	3.150 (2)	150
N3—H3*A*⋯N4^ii^	0.86	2.27	3.065 (2)	153

Symmetry codes: (i) 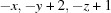; (ii) 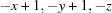.

**Fig. 1.**
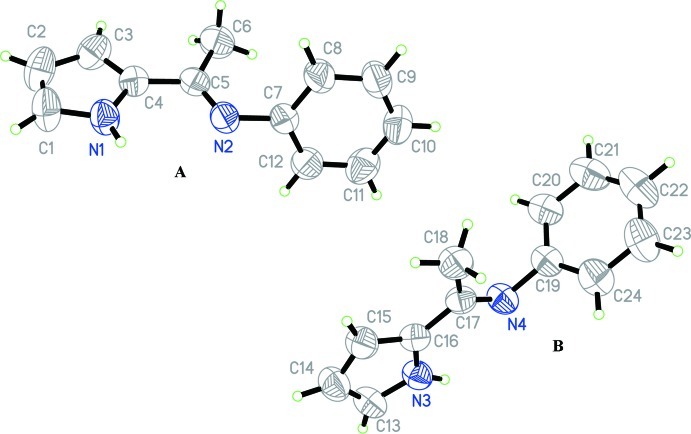
Two independent molecules in the asymmetric unit of the title compound showing the atomic numbering scheme. Displacement ellipsoids are drawn at 50% probability level. H atoms are presented as a small spheres of arbitrary radius.

**Fig. 2.**
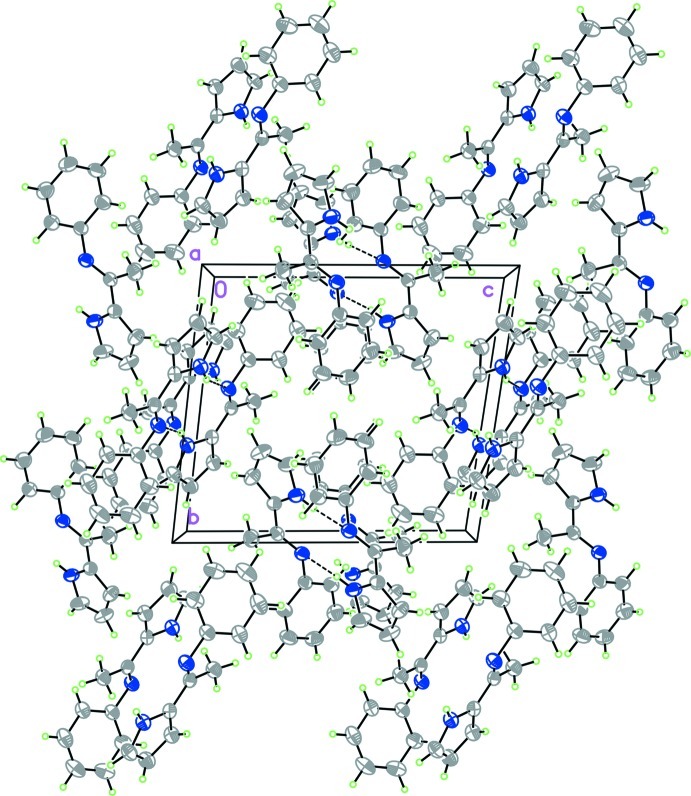
Molecular packing of the title compound with hydrogen bonding shown as dashed lines.

## supplementary crystallographic information

### Comment

In recent years, the bis(imino)pyridine incorporated late transition metal catalysts have received considerable attention because of their antioxidant property and outstanding activity for olefin polymerization (Small *et al.*, 1998; Su *et al.*, 2009**a*,b*). As the five-memberd ring substitute of pyridine six-memberd ring (Matsuo *et al.*, 2001; He *et al.*, 2009), pyrrole was frequently introduced into the skeleton of bis(imino)pyridine ligand to design new ligand and corresponding metal complexes (Britovsek *et al.*, 2003; Dawson *et al.*, 2000). Bis(imino)pyrrole was usually prepared from Schiff base condensation of 2,5-diacetylpyrrole and the aromatic amine (Matsuo *et al.*, 2001). As a contribution to this research field, we report herein the synthesis of mono(imino)pyrrole from 2-acetyl pyrrole and aromatic anime, as well as the crystal structure of the title compound 2-(1-phenyliminoethyl)pyrrole.

The asymmetric unit of the title compound (Fig. 1) comprises of two crystallographically independent molecules *A* and *B*. In each molecule the pyrrole ring and benzene ring are essentially perpendicular, with dihedral angles of 72.37 (7)° and 82.34 (8)° respectively. The benzene ring of molecules *A* are nearly parallel with the benzene ring of molecules *B* with dihedral angle of 14.33 (14)°, but pyrrole ring of molecules *A* forms a comparative large 39.42 (9)° dihedral angle with the pyrrole ring of molecules *B*. On the whole, bond lengths and angles in both molecules are equal in the s.u. range. The crystal packing is stabilized by N–H···N classical intermolecular hydrogen bonds (Table 1, Fig. 2).

### Experimental

The 2-acetyl pyrrole (0.1313 g, 1.20 mmol), aniline (0.1118 g, 1.20 mmol) were placed in a 50 ml flask, after a few drops of acetic acid were added in, the mixture was subjected to radiation in a 800 W microwave oven for 2 min and 3 min on a medium-heat setting. The reaction was monitored by *TLC*, and the crude product was purified by silica gel column chromatography (eluent: petroleum ether/ethyl acetate, 5:1 *v*/*v*), the colourless or light yellow crystals of the title compound were at last obtained by recrystallization from ethanol (yield 0.063 g, 28.4%). M.p. 405.25-406.85 K. The purity and the composition of the compound were checked and characterized by IR spectrum, ^1^H NMR spectrum, mass spectrum, as well as elemental analysis. IR (KBr): ν_C__═__N_ 1653 cm^-1^. ^1^H NMR (400 MHz, CDCl_3_): δ 7.33 (t, H, benzene ring aromatic H), 7.15 (t, 2H, benzene ring aromatic H), 7.02 (d, 2H, benzene ring aromatic H), 6.97 (t, 1H, pyrrole ring aromatic H), 6.33 (d, 1H, pyrrole ring aromatic H), 6.10 (d, 1H, pyrrole ring aromatic H), 2.03 (s, 3H, -N═C(CH_3_)-). MS (EI): m/*z* 184 (*M*). Anal. Calcd for C_12_H_12_N_2_: C, 78.23; H, 6.57; N, 15.21. Found: C, 78.50; H, 7.01; N, 14.96.

Plate like colourless single crystals used in X-ray diffraction studies were grown in ethanolic solution by slow evaporation of the solvent at room temperature.

### Refinement

All H atoms were placed at calculated positions and refined as riding, with C–H = 0.93-0.96Å, N–H = 0.86Å, and with *U*_iso_(H) = 1.2*U*_eq_(C, N) and *U*_iso_(H) = 1.5*U*_eq_(C) for methyl H atoms.

### Crystal data

**Table:**

C_12_H_12_N_2_	*Z* = 4
*M**_r_* = 184.24	*F*(000) = 392
Triclinic, *P*1	*D*_x_ = 1.180 Mg m^−^^3^
Hall symbol: -P 1	Melting point = 405.25–406.85 K
*a* = 8.2236 (14) Å	Mo *K*α radiation, λ = 0.71073 Å
*b* = 11.3306 (19) Å	Cell parameters from 1351 reflections
*c* = 11.913 (2) Å	θ = 2.4–25.1°
α = 95.984 (3)°	µ = 0.07 mm^−^^1^
β = 93.202 (3)°	*T* = 296 K
γ = 109.274 (3)°	Block, colourless
*V* = 1037.3 (3) Å^3^	0.37 × 0.25 × 0.19 mm

### Data collection

**Table:**

Bruker APEXII CCD diffractometer	3655 independent reflections
Radiation source: fine-focus sealed tube	2567 reflections with *I* > 2σ(*I*)
Graphite monochromator	*R*_int_ = 0.023
φ and ω scans	θ_max_ = 25.1°, θ_min_ = 2.4°
Absorption correction: multi-scan (*SADABS*; Bruker, 2008)	*h* = −9→9
*T*_min_ = 0.974, *T*_max_ = 0.987	*k* = −9→13
5277 measured reflections	*l* = −14→11

### Refinement

**Table:**

Refinement on *F*^2^	Secondary atom site location: difference Fourier map
Least-squares matrix: full	Hydrogen site location: inferred from neighbouring sites
*R*[*F*^2^ > 2σ(*F*^2^)] = 0.048	H-atom parameters constrained
*wR*(*F*^2^) = 0.142	*w* = 1/[σ^2^(*F*_o_^2^) + (0.0689*P*)^2^] where *P* = (*F*_o_^2^ + 2*F*_c_^2^)/3
*S* = 1.10	(Δ/σ)_max_ = 0.001
3655 reflections	Δρ_max_ = 0.20 e Å^−^^3^
256 parameters	Δρ_min_ = −0.13 e Å^−^^3^
0 restraints	Extinction correction: *SHELXL97* (Sheldrick, 2008), Fc^*^=kFc[1+0.001xFc^2^λ^3^/sin(2θ)]^-1/4^
Primary atom site location: structure-invariant direct methods	Extinction coefficient: 0.043 (5)

### Special details

**Table:**

Geometry. All s.u.'s (except the s.u. in the dihedral angle between two l.s. planes) are estimated using the full covariance matrix. The cell s.u.'s are taken into account individually in the estimation of s.u.'s in distances, angles and torsion angles; correlations between s.u.'s in cell parameters are only used when they are defined by crystal symmetry. An approximate (isotropic) treatment of cell s.u.'s is used for estimating s.u.'s involving l.s. planes.
Refinement. Refinement of *F*^2^ against ALL reflections. The weighted *R*-factor *wR* and goodness of fit *S* are based on *F*^2^, conventional *R*-factors *R* are based on *F*, with *F* set to zero for negative *F*^2^. The threshold expression of *F*^2^ > σ(*F*^2^) is used only for calculating *R*-factors(gt) *etc*. and is not relevant to the choice of reflections for refinement. *R*-factors based on *F*^2^ are statistically about twice as large as those based on *F*, and *R*-factors based on ALL data will be even larger.

### Fractional atomic coordinates and isotropic or equivalent isotropic displacement parameters (Å^2^)

**Table:**

	*x*	*y*	*z*	*U*_iso_*/*U*_eq_	
N1	0.1204 (2)	1.17200 (13)	0.61426 (13)	0.0613 (4)	
H1A	0.0627	1.1263	0.5534	0.074*	
N2	0.19520 (19)	0.94813 (13)	0.57233 (12)	0.0557 (4)	
N3	0.38995 (19)	0.64751 (14)	−0.01282 (12)	0.0557 (4)	
H3A	0.4486	0.6007	−0.0354	0.067*	
N4	0.31361 (19)	0.43111 (14)	0.10218 (13)	0.0569 (4)	
C1	0.1184 (3)	1.28776 (18)	0.6531 (2)	0.0780 (6)	
H1	0.0539	1.3305	0.6192	0.094*	
C2	0.2261 (3)	1.3311 (2)	0.7498 (2)	0.0812 (7)	
H2	0.2502	1.4089	0.7935	0.097*	
C3	0.2940 (3)	1.23792 (19)	0.77152 (17)	0.0699 (6)	
H3	0.3710	1.2419	0.8332	0.084*	
C4	0.2279 (2)	1.13853 (16)	0.68614 (15)	0.0524 (5)	
C5	0.2640 (2)	1.02211 (16)	0.66444 (14)	0.0519 (5)	
C6	0.3844 (3)	0.9990 (2)	0.75197 (17)	0.0783 (6)	
H6A	0.3216	0.9665	0.8140	0.117*	
H6B	0.4757	1.0767	0.7793	0.117*	
H6C	0.4331	0.9389	0.7187	0.117*	
C7	0.2301 (2)	0.83354 (16)	0.54888 (15)	0.0525 (5)	
C8	0.1630 (3)	0.73360 (18)	0.60871 (18)	0.0726 (6)	
H8	0.0976	0.7418	0.6684	0.087*	
C9	0.1917 (3)	0.62193 (19)	0.5811 (2)	0.0814 (7)	
H9	0.1457	0.5553	0.6224	0.098*	
C10	0.2873 (3)	0.6075 (2)	0.4936 (2)	0.0781 (6)	
H10	0.3072	0.5319	0.4756	0.094*	
C11	0.3536 (3)	0.7066 (2)	0.43257 (19)	0.0772 (6)	
H11	0.4184	0.6980	0.3727	0.093*	
C12	0.3241 (3)	0.81884 (18)	0.46002 (17)	0.0664 (5)	
H12	0.3683	0.8850	0.4179	0.080*	
C13	0.4041 (3)	0.76033 (19)	−0.04693 (17)	0.0651 (5)	
H13	0.4783	0.8000	−0.0982	0.078*	
C14	0.2908 (3)	0.8061 (2)	0.00674 (18)	0.0698 (6)	
H14	0.2736	0.8820	−0.0013	0.084*	
C15	0.2060 (3)	0.71803 (18)	0.07579 (17)	0.0627 (5)	
H15	0.1218	0.7249	0.1225	0.075*	
C16	0.2679 (2)	0.61897 (16)	0.06320 (14)	0.0507 (4)	
C17	0.2239 (2)	0.50444 (16)	0.11674 (14)	0.0503 (4)	
C18	0.0726 (3)	0.47986 (18)	0.18618 (16)	0.0656 (5)	
H18A	−0.0259	0.4157	0.1446	0.098*	
H18B	0.0462	0.5560	0.2025	0.098*	
H18C	0.1006	0.4522	0.2559	0.098*	
C19	0.2680 (2)	0.31984 (17)	0.15675 (16)	0.0561 (5)	
C20	0.3454 (3)	0.3213 (2)	0.26340 (17)	0.0708 (6)	
H20	0.4238	0.3961	0.3014	0.085*	
C21	0.3063 (3)	0.2120 (3)	0.3132 (2)	0.0850 (7)	
H21	0.3581	0.2136	0.3851	0.102*	
C22	0.1926 (3)	0.1014 (2)	0.2583 (2)	0.0873 (7)	
H22	0.1663	0.0283	0.2927	0.105*	
C23	0.1175 (3)	0.0987 (2)	0.1524 (2)	0.0869 (7)	
H23	0.0404	0.0233	0.1147	0.104*	
C24	0.1551 (3)	0.2071 (2)	0.10094 (19)	0.0730 (6)	
H24	0.1042	0.2042	0.0285	0.088*	

### Atomic displacement parameters (Å^2^)

**Table:**

	*U*^11^	*U*^22^	*U*^33^	*U*^12^	*U*^13^	*U*^23^
N1	0.0669 (10)	0.0474 (9)	0.0668 (10)	0.0195 (8)	−0.0074 (8)	0.0011 (7)
N2	0.0601 (9)	0.0484 (8)	0.0593 (9)	0.0191 (7)	0.0030 (8)	0.0102 (7)
N3	0.0549 (9)	0.0621 (10)	0.0579 (9)	0.0271 (7)	0.0110 (7)	0.0155 (7)
N4	0.0530 (9)	0.0618 (9)	0.0627 (9)	0.0236 (8)	0.0156 (7)	0.0197 (7)
C1	0.0800 (15)	0.0517 (12)	0.1002 (17)	0.0274 (11)	−0.0127 (13)	−0.0059 (11)
C2	0.0714 (14)	0.0616 (13)	0.1009 (17)	0.0213 (11)	−0.0030 (13)	−0.0218 (12)
C3	0.0603 (12)	0.0749 (14)	0.0679 (13)	0.0209 (11)	−0.0028 (10)	−0.0087 (11)
C4	0.0485 (10)	0.0518 (10)	0.0555 (11)	0.0150 (8)	0.0054 (8)	0.0070 (8)
C5	0.0497 (10)	0.0543 (11)	0.0520 (10)	0.0155 (8)	0.0071 (8)	0.0141 (9)
C6	0.0889 (16)	0.0862 (15)	0.0665 (13)	0.0400 (13)	−0.0089 (12)	0.0134 (11)
C7	0.0519 (10)	0.0509 (10)	0.0556 (10)	0.0183 (8)	0.0031 (8)	0.0092 (8)
C8	0.0878 (15)	0.0604 (12)	0.0798 (14)	0.0309 (11)	0.0335 (12)	0.0210 (10)
C9	0.1039 (18)	0.0574 (13)	0.0943 (16)	0.0343 (12)	0.0340 (14)	0.0233 (11)
C10	0.0847 (16)	0.0590 (13)	0.0935 (16)	0.0301 (12)	0.0134 (13)	0.0005 (12)
C11	0.0715 (14)	0.0779 (15)	0.0800 (15)	0.0235 (12)	0.0208 (12)	−0.0006 (12)
C12	0.0682 (13)	0.0598 (12)	0.0673 (13)	0.0145 (10)	0.0140 (10)	0.0109 (10)
C13	0.0620 (12)	0.0718 (13)	0.0703 (13)	0.0282 (10)	0.0107 (10)	0.0277 (10)
C14	0.0744 (14)	0.0705 (13)	0.0780 (14)	0.0382 (11)	0.0132 (11)	0.0213 (11)
C15	0.0641 (12)	0.0711 (13)	0.0634 (12)	0.0339 (10)	0.0145 (10)	0.0142 (10)
C16	0.0457 (10)	0.0615 (11)	0.0473 (10)	0.0208 (8)	0.0051 (8)	0.0084 (8)
C17	0.0472 (10)	0.0583 (11)	0.0457 (10)	0.0188 (8)	0.0033 (8)	0.0055 (8)
C18	0.0647 (12)	0.0725 (13)	0.0672 (12)	0.0293 (10)	0.0216 (10)	0.0146 (10)
C19	0.0474 (10)	0.0639 (12)	0.0649 (12)	0.0248 (9)	0.0165 (9)	0.0176 (9)
C20	0.0742 (14)	0.0770 (14)	0.0659 (13)	0.0280 (11)	0.0095 (11)	0.0200 (11)
C21	0.0857 (17)	0.1045 (19)	0.0803 (15)	0.0435 (15)	0.0171 (13)	0.0376 (14)
C22	0.0747 (16)	0.0889 (18)	0.118 (2)	0.0381 (14)	0.0315 (15)	0.0529 (16)
C23	0.0701 (15)	0.0685 (14)	0.121 (2)	0.0177 (11)	0.0122 (14)	0.0271 (14)
C24	0.0618 (13)	0.0717 (14)	0.0846 (15)	0.0196 (11)	0.0001 (11)	0.0195 (11)

### Geometric parameters (Å, º)

**Table:**

N1—C1	1.350 (2)	C10—C11	1.378 (3)
N1—C4	1.365 (2)	C10—H10	0.9300
N1—H1A	0.8600	C11—C12	1.381 (3)
N2—C5	1.286 (2)	C11—H11	0.9300
N2—C7	1.423 (2)	C12—H12	0.9300
N3—C13	1.352 (2)	C13—C14	1.364 (3)
N3—C16	1.371 (2)	C13—H13	0.9300
N3—H3A	0.8600	C14—C15	1.391 (3)
N4—C17	1.286 (2)	C14—H14	0.9300
N4—C19	1.428 (2)	C15—C16	1.376 (2)
C1—C2	1.355 (3)	C15—H15	0.9300
C1—H1	0.9300	C16—C17	1.453 (2)
C2—C3	1.387 (3)	C17—C18	1.498 (2)
C2—H2	0.9300	C18—H18A	0.9600
C3—C4	1.376 (2)	C18—H18B	0.9600
C3—H3	0.9300	C18—H18C	0.9600
C4—C5	1.446 (2)	C19—C24	1.381 (3)
C5—C6	1.500 (3)	C19—C20	1.385 (3)
C6—H6A	0.9600	C20—C21	1.379 (3)
C6—H6B	0.9600	C20—H20	0.9300
C6—H6C	0.9600	C21—C22	1.363 (3)
C7—C12	1.373 (3)	C21—H21	0.9300
C7—C8	1.376 (3)	C22—C23	1.366 (3)
C8—C9	1.373 (3)	C22—H22	0.9300
C8—H8	0.9300	C23—C24	1.381 (3)
C9—C10	1.368 (3)	C23—H23	0.9300
C9—H9	0.9300	C24—H24	0.9300
			
C1—N1—C4	109.71 (17)	C12—C11—H11	119.9
C1—N1—H1A	125.1	C7—C12—C11	120.60 (19)
C4—N1—H1A	125.1	C7—C12—H12	119.7
C5—N2—C7	119.64 (15)	C11—C12—H12	119.7
C13—N3—C16	109.70 (15)	N3—C13—C14	108.30 (18)
C13—N3—H3A	125.2	N3—C13—H13	125.9
C16—N3—H3A	125.2	C14—C13—H13	125.9
C17—N4—C19	118.11 (15)	C13—C14—C15	107.20 (18)
N1—C1—C2	108.44 (19)	C13—C14—H14	126.4
N1—C1—H1	125.8	C15—C14—H14	126.4
C2—C1—H1	125.8	C16—C15—C14	108.33 (17)
C1—C2—C3	107.24 (18)	C16—C15—H15	125.8
C1—C2—H2	126.4	C14—C15—H15	125.8
C3—C2—H2	126.4	N3—C16—C15	106.47 (15)
C4—C3—C2	108.29 (18)	N3—C16—C17	123.18 (15)
C4—C3—H3	125.9	C15—C16—C17	130.35 (16)
C2—C3—H3	125.9	N4—C17—C16	119.49 (16)
N1—C4—C3	106.31 (16)	N4—C17—C18	124.21 (16)
N1—C4—C5	122.82 (16)	C16—C17—C18	116.31 (15)
C3—C4—C5	130.76 (18)	C17—C18—H18A	109.5
N2—C5—C4	118.71 (16)	C17—C18—H18B	109.5
N2—C5—C6	124.98 (17)	H18A—C18—H18B	109.5
C4—C5—C6	116.29 (16)	C17—C18—H18C	109.5
C5—C6—H6A	109.5	H18A—C18—H18C	109.5
C5—C6—H6B	109.5	H18B—C18—H18C	109.5
H6A—C6—H6B	109.5	C24—C19—C20	118.90 (19)
C5—C6—H6C	109.5	C24—C19—N4	120.50 (18)
H6A—C6—H6C	109.5	C20—C19—N4	120.46 (17)
H6B—C6—H6C	109.5	C21—C20—C19	120.0 (2)
C12—C7—C8	118.78 (17)	C21—C20—H20	120.0
C12—C7—N2	119.48 (16)	C19—C20—H20	120.0
C8—C7—N2	121.62 (17)	C22—C21—C20	120.7 (2)
C9—C8—C7	120.61 (19)	C22—C21—H21	119.6
C9—C8—H8	119.7	C20—C21—H21	119.6
C7—C8—H8	119.7	C23—C22—C21	119.7 (2)
C10—C9—C8	120.8 (2)	C23—C22—H22	120.2
C10—C9—H9	119.6	C21—C22—H22	120.2
C8—C9—H9	119.6	C22—C23—C24	120.5 (2)
C9—C10—C11	119.0 (2)	C22—C23—H23	119.7
C9—C10—H10	120.5	C24—C23—H23	119.7
C11—C10—H10	120.5	C19—C24—C23	120.2 (2)
C10—C11—C12	120.2 (2)	C19—C24—H24	119.9
C10—C11—H11	119.9	C23—C24—H24	119.9
			
C4—N1—C1—C2	0.7 (2)	C16—N3—C13—C14	−0.1 (2)
N1—C1—C2—C3	−0.9 (3)	N3—C13—C14—C15	0.2 (2)
C1—C2—C3—C4	0.8 (2)	C13—C14—C15—C16	−0.2 (2)
C1—N1—C4—C3	−0.2 (2)	C13—N3—C16—C15	−0.1 (2)
C1—N1—C4—C5	−176.80 (17)	C13—N3—C16—C17	−179.24 (16)
C2—C3—C4—N1	−0.3 (2)	C14—C15—C16—N3	0.2 (2)
C2—C3—C4—C5	175.84 (19)	C14—C15—C16—C17	179.27 (18)
C7—N2—C5—C4	179.53 (15)	C19—N4—C17—C16	179.47 (15)
C7—N2—C5—C6	1.0 (3)	C19—N4—C17—C18	−0.9 (3)
N1—C4—C5—N2	1.6 (3)	N3—C16—C17—N4	7.2 (3)
C3—C4—C5—N2	−174.05 (19)	C15—C16—C17—N4	−171.75 (18)
N1—C4—C5—C6	−179.77 (17)	N3—C16—C17—C18	−172.52 (16)
C3—C4—C5—C6	4.6 (3)	C15—C16—C17—C18	8.6 (3)
C5—N2—C7—C12	−112.9 (2)	C17—N4—C19—C24	92.6 (2)
C5—N2—C7—C8	71.2 (2)	C17—N4—C19—C20	−91.9 (2)
C12—C7—C8—C9	1.0 (3)	C24—C19—C20—C21	−1.4 (3)
N2—C7—C8—C9	176.9 (2)	N4—C19—C20—C21	−177.05 (18)
C7—C8—C9—C10	−0.1 (4)	C19—C20—C21—C22	0.4 (3)
C8—C9—C10—C11	−0.5 (4)	C20—C21—C22—C23	0.5 (4)
C9—C10—C11—C12	0.2 (3)	C21—C22—C23—C24	−0.4 (4)
C8—C7—C12—C11	−1.2 (3)	C20—C19—C24—C23	1.5 (3)
N2—C7—C12—C11	−177.28 (17)	N4—C19—C24—C23	177.15 (18)
C10—C11—C12—C7	0.6 (3)	C22—C23—C24—C19	−0.7 (3)

### Hydrogen-bond geometry (Å, º)

**Table:**

*D*—H···*A*	*D*—H	H···*A*	*D*···*A*	*D*—H···*A*
N1—H1*A*···N2^i^	0.86	2.38	3.150 (2)	150
N3—H3*A*···N4^ii^	0.86	2.27	3.065 (2)	153

Symmetry codes: (i) −*x*, −*y*+2, −*z*+1; (ii) −*x*+1, −*y*+1, −*z*.
